# LncRNA CASC19 accelerates chondrocytes apoptosis and proinflammatory cytokine production to exacerbate osteoarthritis development through regulating the miR-152-3p/DDX6 axis

**DOI:** 10.1186/s13018-021-02543-x

**Published:** 2021-06-22

**Authors:** Chang Zhou, Tianda He, Liji Chen

**Affiliations:** 1 Department of Orthopedics, The Third Affiliated Hospital of Soochow University, Changzhou, 213000 People’s Republic of China; 2 Department of Osteoarthritis, The Third Affiliated Hospital of Soochow University, Changzhou, 213000 People’s Republic of China; 3 Department of Encephalopathy, Changzhou Hospital of Traditional Chinese Medicine, No. 25 Heping North Road, Changzhou, 213000 People’s Republic of China

**Keywords:** CASC19, miR-152-3p, DDX6, Osteoarthritis

## Abstract

**Background:**

Osteoarthritis (OA) is one kind of degenerative joint disease that happens in articular cartilage and other joint tissues. Long non-coding RNAs (lncRNAs) have been reported to serve as pivotal regulators in many diseases, including OA. However, the role and relevant regulatory mechanisms of CASC19 in OA remain unknown.

**Methods:**

The expression levels of CASC19, miR-152-3p, and DDX6 were identified by reverse-transcription polymerase chain reaction (RT-qPCR). Cell viability and apoptosis were determined by Cell Counting Kit-8 (CCK-8) and flow cytometry assays, respectively. The relationship between miR-152-3p and CASC19 or DDX6 was predicted by bioinformatics tools and verified by the dual-luciferase reporter assay.

**Results:**

CASC19 was verified to exhibit higher expression in OA tissues and cells. Moreover, inhibition of CASC19 weakened proinflammatory cytokine (IL-6, IL-8, and TNF-α) production and cell apoptosis but facilitated cell viability. Experiments of the ceRNA mechanism elucidated that miR-152-3p was a sponge for CASC19, and miR-152-3p targeted DDX6, suggesting that CASC19 sponged miR-152-3p to release DDX6. Finally, results from rescue assays proved that the impacts of CASC19 silencing on chondrocytes apoptosis and proinflammatory cytokine production could be reversed by DDX6 overexpression.

**Conclusions:**

It was concluded that lncRNA CASC19 accelerated chondrocytes apoptosis and proinflammatory cytokine production to exacerbate osteoarthritis development through regulating the miR-152-3p/DDX6 axis. These findings may offer an effective biological target for OA treatment.

## Introduction

Osteoarthritis (OA), one complicated chronic arthropathy characterized by local inflammation and articular cartilage damage and degradation, is one primary cause of disability in the elderly [1, 2]. At present, OA has become a great threat to public health worldwide, and almost 10% of the population and 50% of people aged above 65 suffer from OA [3, 4]. Hence, it is urgent to extend the knowledge of OA pathology and develop more effective therapeutic strategies. Aberrant apoptosis, extracellular matrix, and inflammatory response of chondrocytes are related to cartilage degradation in OA [5, 6]. Therefore, in-depth exploration of chondrocyte-dysfunction-related mechanisms may help to enhance understanding of OA pathogenesis.

Long non-coding RNAs (lncRNAs) are RNAs with more than 200 nucleotides in length and have no protein-coding ability [7–9]. These lncRNAs function as important regulators in multiple diseases, including OA. For example, lncRNA TM1P3 regulates chondrocyte extracellular matrix degradation to participate in OA progression [10]. LncRNA PVT1 absorbs miR-488-3p in OA to modulate chondrocyte apoptosis [11]. LncRNA ZFAS1 regulates cellular processes of chondrocytes in OA [12]. In addition, lncRNA H19 suppression modulates miR-130a in OA to improve LPS-induced damage [13]. CASC19, one of the recently identified lncRNA, has been demonstrated to participate in various cancers. For example, lncRNA CASC19 absorbs miR-301b-3p to modulate LDLR and accelerates non-small cell lung cancer tumorigenesis [14]. LncRNA CASC19/miR-454-3p/RAB5A axis to facilitate glioma progression [15]. Besides, lncRNA CASC19 targets miR-148b/E2F7 axis to aggravate pancreatic cancer progression [16]. LncRNA CASC2 was found to promote OA progression via regulating IL-17 expression [17]. Therefore, we assumed that CASC19, another lncRNA of the cancer susceptibility candidate (CASC) family, might also be involved in OA development. However, the function and regulatory mechanisms of CASC19 in OA are still unclear.

MicroRNAs (miRNAs), another kind of non-coding RNAs with about 18–22 nucleotides, can regulate cell phenotypes, such as cell proliferation, apoptosis, and differentiation [18–20]. Recent investigations have revealed the vital function of miR-152-3p in diseases. For instance, miR-152-3p targets FOXF1 to modulate keloid fibroblast development [21]. Furthermore, miR-152-3p targets CDK8 to regulate hepatic carcinogenesis [22]. CircHIPK3 sponges miR-152-3p to release TGF-β2, thus promoting cardiac fibrosis under hypoxia by regulating fibroblast proliferation and phenotypic switching [23]. Additionally, miR-152 regulates TCF-4 pathway in OA rats to weaken chondrocyte apoptosis and cartilage degeneration [24]. StarBase website predicted that miR-152-3p owned putative complementary sites for CASC19. Nevertheless, the association between miR-152-3p and CASC19 in OA maintains unknown.

In this study, we intended to investigate the functions and potential regulatory mechanism of CASC19 in OA. Findings in this study revealed that CASC19 accelerated chondrocyte apoptosis and proinflammatory cytokine production to exacerbate OA development through regulating miR-152-3p/DDX6 axis, suggesting that CASC19 might be a new target for OA treatment.

## Materials and methods

### Patients and specimens

A total of 40 samples were respectively collected from 20 patients with OA and 20 trauma patients (undergoing lower-extremity amputation) without OA. Patients meeting the diagnostic criteria for OA were included in the study, while patients complicated with other diseases, such as history of joint surgery or rheumatoid arthritis were excluded. The general clinical characteristics of 20 OA patients and 20 trauma patients without OA (control group) were illustrated in Table 1. According to the data acquired, OA and control groups were homogeneous in age, gender, and BMI. For OA patients, total knee arthroplasty was performed and the cartilage of knee joints was taken. For trauma patients, the normal articular cartilage of knee joints was also obtained after lower-extremity amputation according to relevant studies [25, 26]. Informed consent was acquired from all participators. This study was supported by the Human Ethics Committee of Changzhou Hospital of Traditional Chinese Medicine.

**Table 1:** Comparison of clinical characteristics between OA patients and the control group

	OA group (***n*** = 20)	Control group (***n*** = 20)	***P*** value
**Gender**			
Male	11	10	0.64
Female	9	10	
**Age**			
< 60	13	14	0.07
≥ 60	7	6	
**BMI (kg/m**^**2**^**)**			
< 24	7	8	0.12
≥ 24	13	12	
**Kellgren-Lawrence grading**			
2	8		
3	7		
4	5		

### Cell culture

The human chondrocytes C28/I2 cells (ATCC, Manassas, VA) were maintained in Dulbecco’s modified Eagle’s medium (DMEM; Gibco, USA) with 10% fetal bovine serum. All cells were maintained at 37 °C, and 5% CO_2_. For establishing the OA model, C28/I2 cells were stimulated with IL-1β (10 ng/ml).

### Cell transfection

Short hair RNAs (shRNA) against CASC19 (sh-CASC19) and negative control (sh-NC) were bought from GenePharm (Shanghai, China). MiR-152-3p mimics and NC mimics were also acquired from GenePharm. The DDX6 vectors (oe-DDX6) were constructed by cloning its 3′-UTR into the pcDNA3.1 vectors (Thermo Fisher Scientific). The transfection for these vectors was carried out by Lipofectamine 2000 (Thermo Fisher Scientific).

### Reverse-transcription polymerase chain reaction (RT-qPCR)

RNAs were extracted from C28/I2 cells or OA tissues using Trizol reagent (Invitrogen, CA, USA). Synthesis of cDNA was performed with the PrimeScript® RT reagent Kit (Takara, Dalian, China). The PCR was conducted with SYBR Green PCR kit (TaKaRa, Dalian, China) using GAPDH/U6 as endogenous control. The data were processed through the 2^−ΔΔCt^ method.

### Enzyme-linked immunosorbent assay (ELISA)

The inflammatory cytokines interleukin-6 (IL-6), interleukin-8 (IL-8), or tumor necrosis factor-alpha (TNF-α) levels were measured through the corresponding Quantikine ELISA Kits (R&D Systems, Abingdon, UK).

### CCK-8 assay

Cell viability was examined through Cell Counting Kit-8 (CCK-8) (Dojindo, Tokyo, Japan). In short, transfected C28/I2 cells at a density of 1 × 10^4^ cells/well were seeded on 96-well plates. After 0, 24, 48, 72, and 96 h, CCK-8 solution (10 μl) was added into each well, and the cells were incubated for 4 h at room temperature. The cell viability (at 450 nm) was evaluated through the Microplate Reader (Bio-Rad, Hercules, CA, USA).

### Flow cytometry assay

Cell apoptosis was assessed through the Annexin V-FITC Apoptosis Detection Kit (Abcam, Cambridge, UK). Generally, after being rinsed twice with cold PBS solution, C28/I2 cells were resuspended. Afterwards, Annexin V-FITC and propidium iodide (PI) were mixed and incubated. Finally, the apoptosis rate was examined under the flow cytometer (BD Biosciences, San Jose, CA, USA).

### Luciferase reporter assay

For luciferase reporter assay, sequences of CASC19 (or DDX6) were inserted into pmirGLO vectors (Promega, Madison, WI, USA) to produce wild-type CASC19 (or DDX6) vectors (CASC19-Wt or DDX6-Wt). The mutant-type CASC19 (or DDX6) vectors (CASC19-Mut or DDX6-Mut) were also obtained. These vectors with miR-152-3p mimics or NC mimics were co-transfected into C28/I2 cells. About 48 h, the luciferase activity was examined through the Dual-Luciferase reporter assay system (Promega).

### RNA immunoprecipitation (RIP) assay

RIP assay was implemented via Magna RIP RNA-Binding Protein Immunoprecipitation Kit. Cell lysate, RIP buffer, and magnetic beads coupled to Ago2 antibody or normal IgG antibody (as negative control) were mixed. The immunoprecipitated RNA was analyzed through RT-qPCR to quantify gene expression.

### Statistical analysis

Statistical analysis was performed via SPSS 20.0 (SPSS, Inc., Chicago, IL, USA). The data were shown as mean ± SD. Statistical differences were analyzed through Student’s t-test or one-way analysis of variance. P < 0.05 was supposed as statistically significant.

## Results

### CASC19 exhibited higher expression in OA tissues and cells

**Fig. 1:**
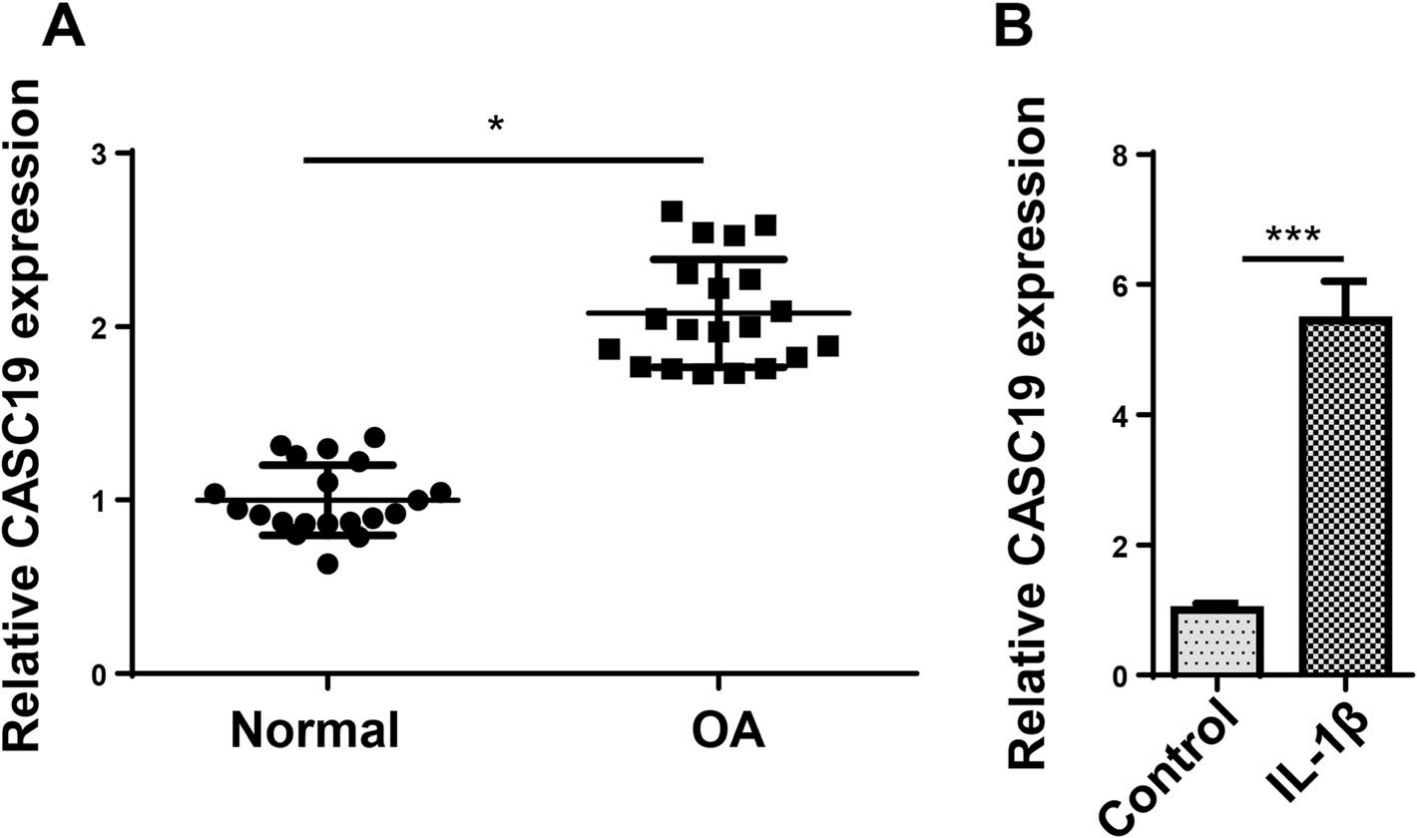
CASC19 exhibited higher expression in OA tissues and OA cell model. **a** The CASC19 expression was tested in OA tissues through RT-qPCR assay. **b** The expression of CASC19 was detected in C28/I2 cells mediated with IL-1β (10 ng/ml) through RT-qPCR. *P < 0.05 and ***P < 0.001

CASC19 has been investigated in various cancers [14–16], but its role in OA keeps unknown. To probe whether CASC19 is altered in OA, RT-qPCR assay was conducted to test CASC19 expression in OA tissues, and the results indicated that CASC19 exhibited higher expression in OA samples (Fig. 1a). Similarly, CASC19 exhibited higher expression in OA cell model (Fig. 1b). To sum up, CASC19 exhibited higher expression in OA tissues and cell model.

### Inhibition of CASC19 inhibited proinflammatory cytokine production and cell apoptosis

**Fig. 2:**
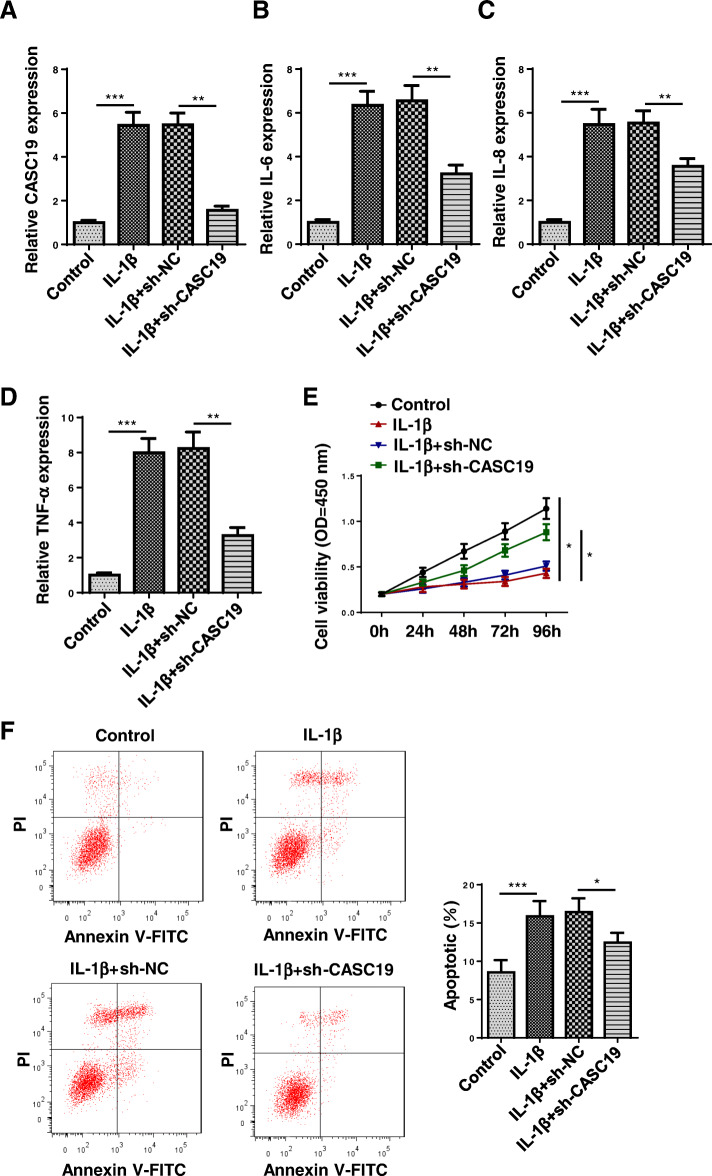
Inhibition of CASC19 weakened proinflammatory cytokine production and cell apoptosis. **a** The knockdown efficiency of CASC19 was verified through RT-qPCR. **b**–**d** The IL-6, IL-8, and TNF-α levels were tested after silencing CASC19 through ELISA assay. **e** The cell viability was assessed after suppressing CASC19 through CCK-8 assay. **f** The cell apoptosis was detected after CASC19 inhibition through flow cytometry assay. *P < 0.05, **P < 0.01, and ***P < 0.001

Next, the function of CASC19 in OA progression was explored. CASC19 expression was markedly reduced after suppressing CASC19 in C28/I2 cells mediated with IL-1β (Fig. 2a). The IL-6, IL-8, and TNF-α levels were reduced through silencing CASC19 (Fig. 2b-d). In addition, the cell viability was increased by CASC19 knockdown (Fig. 2e). Through flow cytometry analysis, it was confirmed that CASC19 suppression retarded cell apoptosis (Fig. 2f). These findings suggested that inhibition of CASC19 inhibited proinflammatory cytokine production and cell apoptosis in OA.

### MiR-152-3p was a sponge for CASC19

**Fig. 3:**
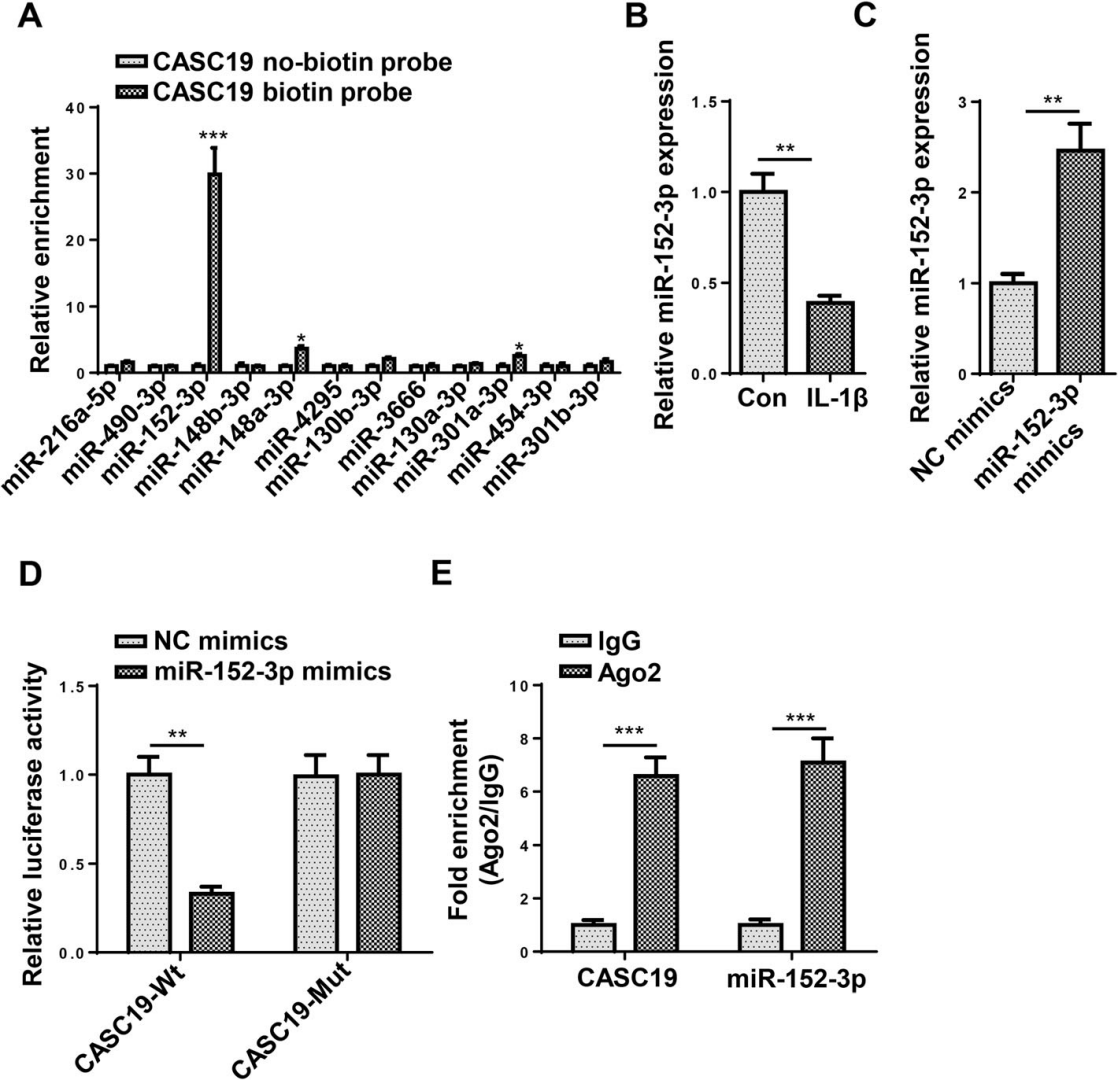
MiR-152-3p was a sponge for CASC19. **a** The miRNAs sponged with CASC19 were predicted through starBase website with the condition of CLIP Data: high stringency (≥ 3). The binding ability between CASC19 and miRNAs was tested by RNA pull-down assay. **b** The miR-152-3p expression was verified through RT-qPCR assay. **c** The overexpression efficiency of miR-152-3p was identified through RT-qPCR assay. **d** The binding ability between CASC19 and miR-152-3p was confirmed through luciferase reporter assay. **e** The binding ability between CASC19 and miR-152-3p was assessed through RIP assay. *P < 0.05, **P < 0.01, and ***P < 0.001

Subsequently, we investigated the ceRNA mechanism related to CASC19. Through StarBase website, several miRNAs were predicted as downstream targets of CASC19 under certain condition (CLIP Data: high stringency (≥ 3)). It was uncovered that miR-152-3p had the strongest binding ability for CASC19 (Fig. 3a). Furthermore, miR-152-3p expression was downregulated in C28/I2 cells triggered with IL-1β (Fig. 3b). The overexpression efficiency of miR-152-3p mimics was verified in Fig. 3c. Findings verified that miR-152-3p overexpression attenuated the luciferase activity of CASC19-Wt vectors but had no effects on CASC19-Mut vectors (Fig. 3d). Additionally, the expression of CASC19 and miR-152-3p was enriched in Ago2 group but not in IgG group, indicating CASC19 sponged miR-152-3p (Fig. 3e). To sum up, miR-152-3p was a sponge for CASC19.

### MiR-152-3p targeted DDX6

**Fig. 4:**
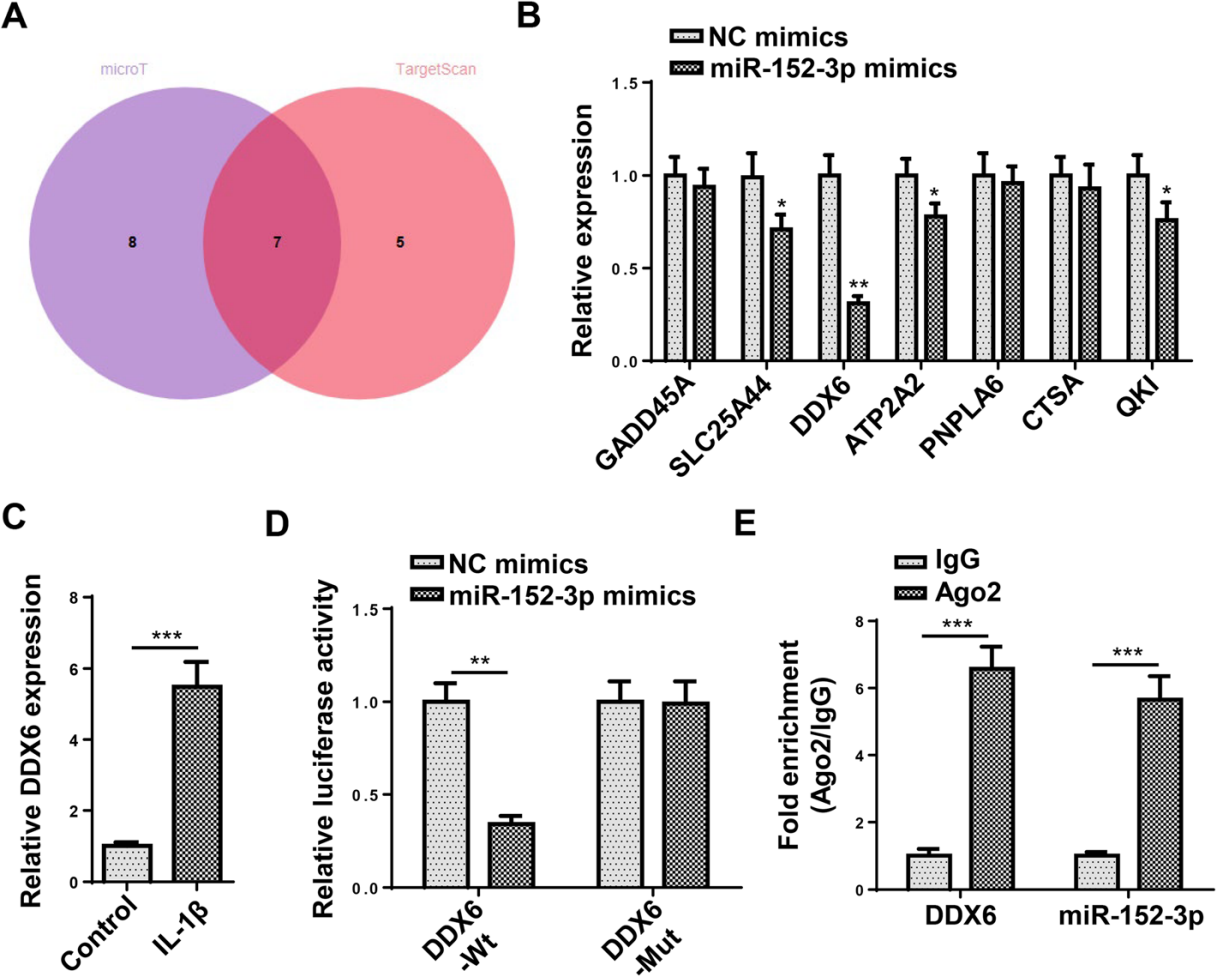
MiR-152-3p targeted DDX6. **a** The Venn diagram displayed these mRNAs which could combine with miR-152-3p. **b** The selected mRNAs expression was detected after overexpressing miR-152-3p. **c** The DDX6 expression was examined through RT-qPCR assay. **d** The binding capacity between miR-152-3p and DDX6 was tested through luciferase reporter assay. **e** The interaction between miR-152-3p and DDX6 was evaluated through RIP assay. **P < 0.01 and ***P < 0.001

In the next step, the potential mRNAs which could combine with miR-152-3p were investigated. The Venn diagram illustrated that 7 mRNAs (GADD45A, SLC25A44, DDX6, ATP2A2, PNPLA6, CTSA, and QKI) all existed in microT and TargetScan database (Fig. 4a). Among these mRNAs, DDX6 expression showed the largest decrease after miR-152-3p overexpression, thus DDX6 was selected for further study (Fig. 4b). Moreover, RT-qPCR indicated that DDX6 expression was upregulated in C28/I2 cells mediated with IL-1β (Fig. 4c). In addition, it was uncovered that miR-152-3p overexpression attenuated the luciferase activity of DDX6-Wt vectors but had no effects on DDX6-Mut vectors (Fig. 4d). Furthermore, the abundance of miR-152-3p and DDX6 was found in Ago2 group, suggesting miR-152-3p targeted DDX6 (Fig. 4e). To sum up, miR-152-3p combined with DDX6.

### CASC19 regulated proinflammatory cytokine production and cell apoptosis through DDX6

**Fig. 5:**
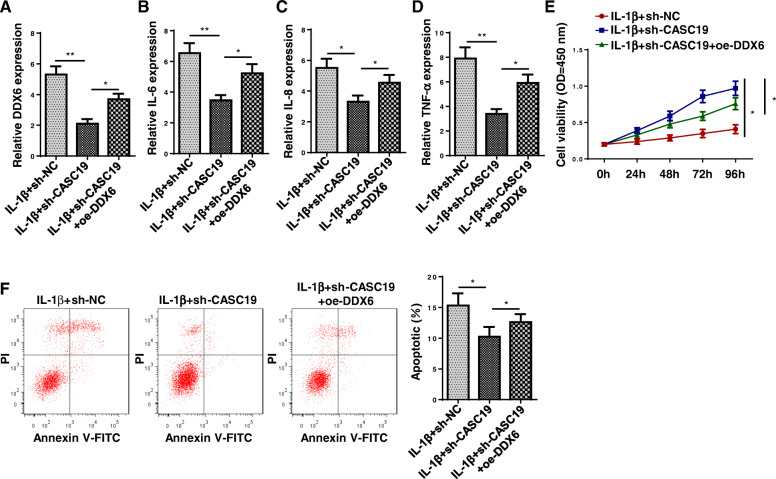
CASC19 regulated proinflammatory cytokine production and cell apoptosis through DDX6. Groups were divided into the IL-1β+sh-NC, IL-1β+sh-CASC19, and IL-1β+sh-CASC19+oe-DDX6 group. **a** The overexpression efficiency of DDX6 was notarized through RT-qPCR assay. **b**–**d** The IL-6, IL-8, and TNF-α levels were tested through ELISA assay. **e** The cell viability was assessed through CCK-8 assay. **f** The cell apoptosis was examined through flow cytometry assay. *P < 0.05 and **P < 0.01

To explore whether CASC19 regulated proinflammatory cytokine production and cell apoptosis through DDX6, rescue assays were performed. The upregulated expression of DDX6 was verified after overexpressing DDX6 (Fig. 5a). The reduced IL-6, IL-8, and TNF-α levels caused by repressing CASC19 could be reversed by upregulating DDX6 (Fig. 5b-d). As displayed in Fig. 5e, overexpression of DDX6 could offset the increased cell viability induced by CASC19 inhibition. In addition, the inhibitive effects of CASC19 suppression on cell apoptosis could be rescued by DDX6 overexpression (Fig. 5f). These results suggested that CASC19 regulated proinflammatory cytokine production and cell apoptosis through DDX6.

## Discussion

With the fast development of science and technology, an increasing number of lncRNAs have been identified as crucial regulators in various diseases [27, 28]. CASC19 has been uncovered to be implicated in various cancers [14–16], but its function in OA remains indistinct. In this work, CASC19 exhibited higher expression in OA tissues and cell model. Moreover, inhibition of CASC19 weakened proinflammatory cytokine production and cell apoptosis.

LncRNAs are confirmed to be one pivotal player in competing endogenous RNA (ceRNA)-mediated mechanisms because they can act as sponges for miRNAs to regulate mRNA expression [29–31]. Interestingly, this regulatory mechanism is also widely involved in OA progression. For instance, lncRNA NEAT1/miR-181a/GPD1L axis regulates chondrocyte proliferation, apoptosis, and inflammation [32]. In addition, lncRNA TUG1/miR-195/MMP-13 axis facilitates the degradation of chondrocyte extracellular matrix induced by OA [33]. LncRNA SNHG7 absorbs miR-214-5p to modulate PPARGC1B pathways and improves IL-1β-induced OA [34]. Moreover, lncRNA MEG3 targets miR-93/TGFBR2 axis to retard extracellular matrix degradation in OA [35]. Through StarBase website, miR-152-3p was predicted to be a sponge for CASC19, and DDX6 was a downstream mRNA of miR-152-3p. Additionally, luciferase reporter and RIP assays elucidated that CASC19 absorbed miR-152-3p to release DDX6.

It has been reported that DEAD-box protein 6 (DDX6) plays an important role in various diseases. For example, RNA helicase DDX6 upregulates c-Myc expression by serving as an oncogene in gastric cancer [36]. Additionally, DDX6 exhibited higher expression and modulated by miR-124 in colon cancer [37]. MiR-130 family modulates P-body protein DDX6 to modulate the hypoxia response signal [38]. In our study, results from rescue assays certified that DDX6 overexpression could reverse the effects of CASC19 suppression on chondrocytes apoptosis and proinflammatory cytokine production.

## Conclusions

This study was the first to discover the function and related ceRNA regulatory mechanism of CASC19 in OA. Our findings revealed that CASC19 accelerated chondrocytes apoptosis and proinflammatory cytokine production to exacerbate OA development through regulating miR-152-3p/DDX6 axis, indicating CASC19 may be a promising target for OA treatment and providing a novel direction of improving OA therapeutic methods for orthopedists. Although our study highlighted a CASC19-regulated regulatory mechanism in osteoarthritis progression, it is still limited because of the small sample size. In the future, this study will be further improved by expanding the sample size and adding more in vitro and in vivo experiments.

## Data Availability

The datasets used and/or analyzed during the current study are available from the corresponding author on reasonable request.

## Abbreviations

lncRNAs: Long non-coding RNAs
OA: Osteoarthritis
RT-qPCR: Reverse-transcription polymerase chain reaction
CCK-8: Cell Counting Kit-8
shRNA: Short hair RNAs
miRNA: MicroRNA
DDX6: DEAD-box protein 6
FITC: Annexin V-fluorescein isothiocyanate
PI: Propidium iodide
Wt: Wild-type
Mut: Mutant
ELISA: Enzyme-linked immunosorbent assay
IL-6: Interleukin-6
IL-8: Interleukin-8
TNF-α: Tumor necrosis factor-alpha
